# Donor sperm IVF pregnancies exhibit elevated risk of new-onset hypertensive disorders: a retrospective cohort study

**DOI:** 10.1007/s10815-026-03834-0

**Published:** 2026-02-24

**Authors:** Binbin Tu, Mengjie Fan, Ping Liu, Rong Li, Jie Qiao, Rui Yang

**Affiliations:** 1 Center for Reproductive Medicine, Department of Obstetrics and Gynecology, Peking University Third Hospital, Beijing, China; 2 National Clinical Research Center for Obstetrics and Gynecology, Peking University Third Hospital, Beijing, China; 3 Present Address: Key Laboratory of Assisted Reproduction (Peking University), Ministry of Education, Beijing, China; 4 Beijing Key Laboratory of Reproductive Endocrinology and Assisted Reproductive Technology, Peking University Third Hospital, Beijing, China; 5 State Key Laboratory of Female Fertility Promotion, Department of Obstetrics and Gynecology, Peking University Third Hospital, Beijing, China

**Keywords:** Donor sperm, Partner sperm, In vitro fertilization, Hypertensive disorders of pregnancy

## Abstract

**Purpose:**

To evaluate the incidence of new-onset Hypertensive Disorders of Pregnancy (HDP) in donor sperm IVF (DS-IVF) compared with partner sperm IVF (PS-IVF) pregnancies.

**Methods:**

We retrospectively analyzed a total of 855 DS-IVF cycles and 4,816 PS-IVF cycles of pregnancies delivering after 20 weeks’ gestation achieved live birth between January 2010 and December 2022. All patients underwent fresh embryo transfer after oocyte retrieval. The incidence of new-onset HDP was compared between the two groups.

**Results:**

The overall incidence of new-onset HDP was 1.7%, with rates of 2.7% in DS-IVF group and 1.6% in PS-IVF group. Multivariate Analysis of risk factors in the entire cohort identified twin pregnancy (OR, 2.483: 95%CI: 1.649–3.739, *P* < 0.001), elevated body mass index (BMI) (OR, 1.150: 95%CI: 1.095–1.209, *P* < 0.001), and the use of donor sperm (OR, 2.063: 95%CI: 1.229–3.463, *P* = 0.006) as independent risk factors for new-onset HDP. In subgroup analyses of both DS-IVF and PS-IVF cohorts, twin pregnancy [DS-IVF: OR, 2.575: 95%CI: 1.085–6.115, *P* = 0.032; PS-IVF: OR, 2.454: 95%CI: 1.542–3.906, *p* < 0.001] and elevated BMI [DS-IVF: OR, 1.152: 95%CI: 1.026–1.292, *P* = 0.016; PS-IVF: OR, 1.150: 95%CI: 1.089–1.215, *p* < 0.001] were identified as independent risk factors for new-onset HDP.

**Conclusion:**

The use of donor sperm is an independent risk factor for new-onset HDP in IVF-conceived pregnancies.

## Introduction

Donor sperm in vitro fertilization (DS-IVF) has become a critical solution for couples facing severe male infertility, genetic disorders, or for single women and same-sex female partners seeking conception. The utilization of donor sperm in assisted reproductive technology (ART) has steadily increased in recent years, in line with the latest clinical practice guidelines for gamete donation [1]. In the United States, donor sperm was utilized in approximately 6% of all ART cycles in 2014——predominantly in IVF (> 99%), along with a small proportion in gamete intrafallopian transfer (GIFT) and zygote intrafallopian transfer (ZIFT) procedures [2], and this proportion has risen to 8.8% in 2022 according to the latest national ART surveillance data, reflecting a continuous growth trend in clinical application [3].

While DS-IVF demonstrates comparable live birth rates to partner sperm IVF (PS-IVF) [2, 4], its distinct biological implications-particularly the introduction of non-self paternal antigens-warrant further investigation into its long-term maternal and fetal outcomes. The “paternal antigen tolerance hypothesis” proposes that repeated exposure to partner semen induces maternal immune adaptation, promoting trophoblast invasion and spiral artery remodeling [5]. Such prior repeated semen exposures from a single male individual are, however, absent in donor sperm conceptions compared with conceptions following use of partner sperm, as in the case of pregnancy following change of partner [6], which leads to hypertensive disorders of pregnancy (HDP), including pregnancy-induced hypertension (PIH) and preeclampsia (PE). Previous studies on donor sperm-assisted pregnancies, including intrauterine insemination (IUI) and IVF, have yielded inconsistent findings. Some studies suggest a higher incidence of hypertensive disorders and placental dysfunction in donor sperm pregnancies compared to partner sperm conceptions [7], while others report no significant differences [8, 9]. However, these studies predominantly focus on IUI, with limited data specific to DS-IVF, leaving a gap in understanding its distinct immunological and perinatal implications in DS-IVF.

Therefore, our study aims to address the gap in knowledge by retrospectively comparing the incidence of HDP between DS-IVF and PS-IVF pregnancies. By elucidating potential risks and underlying mechanisms, our findings may inform clinical counseling and optimize perinatal care for donor-conceived pregnancies.

## Materials and methods

### Study design and population

This retrospective study was conducted at the Reproductive Center of Peking University Third Hospital (Beijing, China). The study was approved by the Institutional Research Board of Peking University Third Hospital (Approval No. 2018SZ-002). Patients’ data for this study were obtained from the Clinical Reproductive Medicine Management System/Electronic Medical Record Cohort Database. Pregnancy outcomes, as well as obstetric and neonatal outcomes, were collected through telephone follow-ups or retrieved from the obstetric medical record system of the hospital. All diagnostic information related to HDP was extracted from the obstetric medical record system and defined based on the criteria established by the American College of Obstetricians and Gynecologists (ACOG).

The inclusion criteria were summarized as follows: female age between 20 and 45 years; pregnancies delivering after 20 weeks’ gestation achieved live birth that resulted from DS-IVF or PS-IVF (indicated for tubal factor infertility) between January 2010 and December 2022. DS-IVF refers to cycles utilizing cryopreserved sperm from anonymous donors, while PS-IVF denotes cycles using fresh sperm from the female partner's spouse. All patients underwent fresh embryo transfer after oocyte retrieval. The exclusion criteria were as follows: patients with polycystic ovary syndrome (PCOS); patients with recurrent pregnancy loss (RPL) [10] or antiphospholipid syndrome (APS) [11]; patients with other diseases, including diabetes and kidney diseases; history of smoking; incomplete important data. Notably, two critically important exclusion criteria were chronic hypertension and history of gestational hypertension, given their significant impact on the study population stratification. The definition of PCOS followed the Rotterdam criteria [12], requiring the fulfillment of more than 2 of the following items: oligo- or anovulation; clinical or biochemical signs of hyperandrogenism; and polycystic ovaries, with the exclusion of other etiologies (e.g., congenital adrenal hyperplasia, androgen-secreting tumors, or Cushing’s syndrome).

### Outcomes

New-onset HDP is defined as the first occurrence of hypertension in pregnant women with no prior history of hypertension or HDP. The diagnosis of new-onset HDP was based on the criteria established by ACOG, encompassing gestational hypertension, preeclampsia, and eclampsia. According to ACOG, gestational hypertension is defined as a systolic blood pressure (SBP) ≥ 140 mmHg or diastolic blood pressure (DBP) ≥ 90 mmHg occurring after 20 weeks of gestation in a previously normotensive patient, without proteinuria or other systemic findings indicative of preeclampsia. Preeclampsia is diagnosed when hypertension is accompanied by proteinuria (≥ 300 mg in a 24-h urine collection or a protein/creatinine ratio ≥ 0.3) or in the absence of proteinuria, by the presence of severe features, such as thrombocytopenia, impaired liver function, renal insufficiency, pulmonary edema, or new-onset cerebral or visual disturbances. Hemolysis, elevated liver enzymes, and low platelet count (HELLP) syndrome is a severe form of preeclampsia characterized by a combination of Hemolysis (H), Elevated Liver Enzymes (EL), and Low Platelet Count (LP). Eclampsia, a severe complication of preeclampsia, involves the occurrence of seizures not attributable to other causes [13].

### Statistical analysis

Statistical analysis was carried out using SPSS 27.0 software (IBM Corp., Armonk, NY, USA). The differences between groups were analyzed by independent sample t-tests if data were normally distributed; otherwise, they were analyzed by Kruskal–Wallis test. Categorical variables are presented as frequency and percentage, and they were analyzed using Chi-square test. The PS-IVF group was taken as the reference group. Multivariate logistic regression analysis was applied to assess new-onset HDP-related confounding factors. The level of statistical significance was defined as *P* < 0.05.

## Results

### Patient and baseline characteristics

The study enrolled a total of 855 DS-IVF cycles and 4,816 PS-IVF cycles. The baseline demographic and clinical characteristics of the entire cohort, as well as DS-IVF and PS-IVF groups, are summarized in Table 1. DS-IVF group demonstrated significantly younger female age (30.58 ± 3.72 vs. 31.80 ± 4.01 years, *P* < 0.001), younger male age (31.88 ± 4.37 vs. 33.16 ± 4.90 years, *P* < 0.001), shorter duration of infertility (4.05 ± 3.09 vs. 4.33 ± 3.31 years, *P* = 0.019), and fewer previous pregnancies (0.16 ± 0.48 vs. 1.05 ± 1.18, *P* < 0.001) compared to PS-IVF group. Notably, the proportion of patients with primary infertility was also significantly elevated in the donor sperm IVF group (87.1% vs. 38.9%, *P* < 0.001). Other variables, such as BMI, Anti-Müllerian hormone (AMH) and whether twin pregnancy, did not differ significantly between groups.

**Table 1:** Baseline demographic and clinical characteristics of cycles with livebirth after DS-IVF and PS-IVF

	Overall (*n* = 5671)	Donor sperm IVF (*n* = 855)	Partner sperm IVF (*n* = 4816)	t/X^2^	P
Maternal Age (years)	31.61 ± 4.00	30.58 ± 3.72	31.80 ± 4.01	8.748	< 0.001
Male age (years)	32.96 ± 4.85	31.88 ± 4.37	33.16 ± 4.90	7.729	< 0.001
Body mass index (kg/m^2^)	22.50 ± 3.30	22.52 ± 3.27	22.49 ± 3.31	−0.264	0.792
Duration of infertility (years)	4.28 ± 3.28	4.05 ± 3.09	4.33 ± 3.31	2.354	0.019
No. of previous pregnancies	0.91 ± 1.15	0.16 ± 0.48	1.05 ± 1.18	37.435	< 0.001
Anti-Müllerian hormone (ng/ml)	2.88 ± 2.04	2.93 ± 2.03	2.85 ± 2.05	−0.628	0.530
Type of infertility, n (%)				678.727	< 0.001
Primary infertility	2620(46.2)	745(87.1)	1875(38.9)		
Secondary infertility	3051(53.8)	110(12.9)	2941(61.1)		
Twin pregnancy, n (%)				0.000	0.999
Yes	1645(29.0)	248(29.0)	1397(29.0)		
No	4026(71.0)	607(71.0)	3419(71.0)		
New-onset HDP, n (%)				5.234	0.022
Yes	99(1.7)	23(2.7)	76(1.6)		
No	5572(98.3)	832(97.3)	4740(98.4)		

### Incidence of new-onset HDP

The incidence of new-onset HDP was 1.7% in the entire cohort, with rates of 2.7% in the DS-IVF group and 1.6% in the PS-IVF group (Table 1). Notably, despite significantly younger maternal age in the DS-IVF cohort compared to the PS-IVF group (*P* < 0.001), the incidence of new-onset HDP remained significantly higher in the former (*P* = 0.022).

### Risk factors of new-onset HDP for all IVF pregnancies

As shown in Table 2, among the entire IVF cohort, univariable analysis identified several significant risk factors associated with new-onset HDP. BMI was significantly higher in the HDP group compared to the non-HDP group (24.40 ± 3.55 vs. 22.46 ± 3.29 kg/m^2^, *P* < 0.001). The proportion of twin pregnancy was higher in the HDP group compared to the non-HDP group (47.5% vs. 28.7%, *P* < 0.001). Additionally, donor IVF was more prevalent in the HDP group compared to the non-HDP group (23.2% vs 14.9%, *p* = 0.022). The risk Factors demonstrating statistical significance in univariate regression analysis, along with factors which were known widely to be associated with HDP (e.g., maternal age, gravidity), were simultaneously incorporated into the multivariate model. The analysis identified twin pregnancy (OR, 2.483: 95%CI: 1.649–3.739, *P* < 0.001), elevated BMI (OR, 1.150: 95%CI: 1.095–1.209, *P* < 0.001), and the use of donor sperm (OR, 2.063: 95%CI: 1.229–3.463, *P* = 0.006) as independent risk factors for new-onset HDP (Table 3).

**Table 2:** Univariable analysis of factors associated with new-onset HDP in the overall IVF cycles

	Non new-onset HDP (*n* = 5572)	New-onset HDP (*n* = 99)	t/X^2^	*P*
Maternal Age (years)	31.60 ± 3.99	32.20 ± 3.73	−1.479	0.139
Male age (years)	32.96 ± 4.85	33.51 ± 4.65	−1.119	0.263
Body mass index (kg/m^2^)	22.46 ± 3.29	24.40 ± 3.55	−5.783	< 0.001
Duration of infertility (years)	4.28 ± 3.28	4.38 ± 3.38	−0.294	0.769
No. of previous pregnancies	0.91 ± 1.15	1.07 ± 1.33	−1.181	0.240
Anti-Müllerian hormone (ng/ml)	2.89 ± 2.05	2.64 ± 1.83	0.726	0.468
Type of infertility, n (%)			0.003	0.958
Primary infertility	2574(46.2)	46(46.5)		
Secondary infertility	2998(53.8)	53(53.5)		
Twin pregnancy, n (%)			16.687	< 0.001
Yes	1598(28.7)	47(47.5)		
No	3974(71.3)	52(52.5)		
Donor sperm, n (%)			5.234	0.022
Yes	832(14.9)	23(23.2)		
No	4740(85.1)	76(76.8)

**Table 3:** Multivariable analysis of new-onset HDP occurrence in the overall IVF cycles

	OR (95%CI)	*P*
Maternal Age (years)	1.053(0.999–1.110)	0.054
Body mass index (kg/m^2^)	1.150(1.095–1.209)	< 0.001
No. of previous pregnancies	1.150(0.976–1.355)	0.094
Twin pregnancy		
Yes	2.483(1.649–3.739)	< 0.001
No	Ref	
Donor sperm		
Yes	2.063(1.229–3.463)	0.006
No	Ref	

### Risk factors of new-onset HDP for DS-IVF pregnancies

As shown in Table 4, among DS-IVF cohort, univariable analysis identified BMI and twin pregnancy associated with new-onset HDP. BMI was significantly higher in the HDP group compared to the non-HDP group (24.25 ± 3.94 vs. 22.52 ± 3.27 kg/m^2^, *p* = 0.012). The proportion of twin pregnancy was higher in the HDP group compared to the non-HDP group (47.8% vs. 28.5%, *p* < 0.001). The risk factors demonstrating statistical significance in univariate regression analysis, along with factors known widely to be associated with HDP (e.g., maternal age, gravidity), were incorporated into the multivariate model. The analysis identified twin pregnancy (OR, 2.575: 95%CI: 1.085–6.115, *P* = 0.032) and elevated BMI (OR, 1.152: 95%CI: 1.026–1.292, *P* = 0.016) as independent risk factors for HDP (Table 5).

**Table 4:** Univariable analysis of factors associated with new-onset HDP in DS-IVF cycles

	Non new-onset HDP (*n* = 832)	New-onset HDP (*n* = 23)	t/X^2^	*P*
Maternal Age (years)	30.57 ± 3.72	30.70 ± 3.57	−0.154	0.878
Male age (years)	31.86 ± 4.39	32.43 ± 3.70	−0.618	0.537
Body mass index (kg/m^2^)	22.52 ± 3.27	24.25 ± 3.94	−2.524	0.012
Duration of infertility (years)	4.04 ± 3.10	4.35 ± 2.85	−0.443	0.658
No. of previous pregnancies	0.16 ± 0.48	0.17 ± 0.49	−0.138	0.891
Anti-Müllerian hormone (ng/ml)	2.92 ± 2.03	3.45 ± 2.00	0.926	0.355
Type of infertility, n (%)			0.001	0.979
Primary infertility	725(87.1)	20(87.0)		
Secondary infertility	107(12.9)	3(13.0)		
Twin pregnancy, n (%)			4.065	0.044
Yes	237(28.5)	11(47.8)		
No	595(71.5)	12(52.2)

**Table 5:** Multivariable analysis of new-onset HDP occurrence in DS-IVF cycles

	OR(95%CI)	*P*
Maternal Age (years)	1.043(0.926–1.175)	0.491
Body mass index (kg/m^2^)	1.152(1.026–1.292)	0.016
No. of previous pregnancies	0.861(0.337–2.201)	0.755
Twin pregnancy		
Yes	2.575(1.085–6.115)	0.032
No	Ref	

### Risk factors of new-onset HDP for PS-IVF pregnancies

As shown in Table 6, among PS-IVF cohort, univariable analysis identified BMI and twin pregnancy associated with new-onset HDP. BMI was significantly higher in the HDP group compared to the non-HDP group (24.44 ± 3.46 vs. 22.46 ± 3.29 kg/m^2^, *p* < 0.001). The proportion of twin pregnancy was higher in the HDP group compared to the non-HDP group (47.4% vs. 28.7%, *p* < 0.001). The risk factors demonstrating statistical significance in univariate regression analysis, along with factors known widely to be associated with HDP (e.g., maternal age, gravidity), were incorporated into the multivariate model. The analysis identified twin pregnancy (OR, 2.454: 95%CI: 1.542–3.906, *p* < 0.001) and elevated BMI (OR, 1.150: 95%CI: 1.089–1.215, *p* < 0.001) as independent risk factors for HDP (Table 7).

**Table 6:** Univariable analysis of factors associated with new-onset HDP in PS-IVF cycles

	Non new-onset HDP (*n* = 4740)	New-onset HDP (*n* = 76)	t/X^2^	*p*
Maternal Age (years)	31.79 ± 4.01	32.66 ± 3.67	−1.885	0.059
Male age (years)	33.15 ± 4.90	33.83 ± 4.87	−1.204	0.229
Body mass index (kg/m^2^)	22.46 ± 3.29	24.44 ± 3.46	−4.922	< 0.001
Duration of infertility (years)	4.32 ± 3.31	4.39 ± 3.53	−0.173	0.862
No. of previous pregnancies	1.04 ± 1.18	1.34 ± 1.39	−1.862	0.066
Anti-Müllerian hormone (ng/ml)	2.88 ± 2.06	2.16 ± 1.58	1.616	0.107
Type of infertility, n (%)			0.724	0.395
Primary infertility	1849(39.0)	26(34.2)		
Secondary infertility	2891(61.0)	50(65.8)		
Twin pregnancy, n (%)			12.641	< 0.001
Yes	1361(28.7)	36(47.4)		
No	3379(71.3)	40(52.6)

**Table 7:** Multivariable analysis of new-onset HDP occurrence in PS-IVF cycles

	OR(95%CI)	*P*
Maternal Age (years)	1.055(0.095–1.119)	0.072
Body mass index (kg/m^2^)	1.150(1.089–1.215)	< 0.001
No. of previous pregnancies	1.162(0.984–1.373)	0.077
Twin pregnancy		
Yes	2.454(1.542–3.906)	< 0.001
No	Ref	

## Discussion

This analysis of the overall cohort revealed significantly elevated BMI, twin pregnancy and donor sperm utilization in the HDP group versus the non-HDP group. Logistic regression established donor sperm utilization as an independent risk factor for new-onset HDP. Among pregnancies conceived using donor sperm and partner sperm independently, higher BMI and twin pregnancy were independent risk factors for new-onset HDP.

Our findings demonstrated donor sperm conception as an independent risk factor for new-onset HDP. This finding is consistent to previous studies, which have reported sperm donation to be associated with a significant increase incidence of HDP. While donor sperm is utilized in both IUI and IVF, the majority of existing literature has focused on outcomes related to IUI. Previous studies have reported a higher incidence of preeclampsia following donor sperm insemination compared with partner sperm insemination or spontaneous conceptions [14–16]. Evidence from multiple meta-analyses also provides stronger supporting evidence for a greater risk of HDP in pregnancies achieved through donor sperm IUI versus partner sperm IUI. One meta-analysis [17] suggested a 60% increased risk of preeclampsia associated with sperm donation. Another meta-analyses[8] revealed that IUI with donor sperm was associated with a significantly elevated risk of preeclampsia (pooled adjusted odds ratio [aOR] 1.77, 95% CI 1.26–2.48) and HDP (pooled aOR 1.55, 95%CI 1.20–2.00) compared to IUI using partner sperm. Allen et al. [18]reported an increase relative risk of both combined hypertensive disorders of pregnancy and preeclampsia in pregnancies conceived with donor sperm compared to those conceived with partner sperm.

Literature comparing DS-IVF and PS-IVF remains scant. A study based on 741 families from 18 fertility clinics [19] reported a higher incidence of high blood pressure during pregnancy in homologous IVF than sperm donation IVF (13.1% vs 10.8%). However, some studies have yielded conflicting results. An Australian statewide retrospective cohort study of 1435 donor sperm-conceived pregnancies [9] found that the rate of hypertensive disorders was 6.1% compared to 5.2% among controls, with an unadjusted odds ratio (OR) of 1.18 (95% CI 0.93–1.49). After adjusting for covariates (maternal age, BMI and ICSI), no difference remained in the risk of hypertensive disorders of pregnancy between donor sperm and partner-sperm-conceived IVF pregnancies (aOR 0.94; 95% CI 0.73–1.21). A meta-analysis [8] also demonstrated no statistically significant differences in the incidence of HDP, preeclampsia, or preterm birth between donor and partner sperm IVF cycles.

DS-IVF pregnancy was established as an independent risk factor for new-onset HDP in our study. This result aligns with the immunological theory underpinning HDP [20, 21], which proposes that exposure to novel antigenic stimuli (as occurs with donor gametes, sperm or oocytes) disrupts maternal immunological tolerance, potentially disrupting trophoblast invasion and spiral artery remodeling—a hallmark of preeclampsia pathogenesis. The lack of immune tolerance due to changing paternal antigens was suggested as the possible reason for the higher new-onset HDP reported in earlier studies following use of donor sperm compared with partner sperm ART [5]. Unlike partner-derived sperm, donor sperm carry foreign human leukocyte antigen (HLA) profiles and surface proteins, potentially disrupting immune tolerance mechanisms critical for successful implantation and placental development. Such immunological mismatches could theoretically impair trophoblast invasion, spiral artery remodeling, or provoke subclinical inflammation, thereby increasing susceptibility to pregnancy complications like pregnancy-induced hypertension and pre-eclampsia [22]. The following observations provide additional support for the aforementioned conclusions and hypotheses. Dekker et al. [23] proposed the "paternal antigen exposure timing" hypothesis to explain the increased risk of HDP observed in pregnancies with a new partner [24], or after short duration of exposure to partner sperm [25], or after a long interval between pregnancies [26]. This effect of less exposure to antigens and higher risk of preeclampsia may also explain that the fewer the number of IUI-D cycles per woman, the higher the risk of PE [15].

As previous studies have established that both maternal age and parity are associated with the incidence of HDP, we included these variables in the multivariate analysis in our study. This was done to minimize the potential confounding effects, even though univariate analysis revealed no significant association between maternal age or parity and hypertensive disorders in all IVF cycles, DS-IVF cycles, or PS-IVF cycles. We found that, besides donor sperm usage, elevated BMI and twin pregnancy remained independent risk factors for hypertensive disorders in pregnancy, which was consistent with previous research. As DS-IVF cycles typically involve women with normal fertility, we restricted the patient cohort in PS-IVF cycles to those with tubal factor infertility. This selection strategy was implemented to minimize the heterogeneity of the study population and ensure valid comparisons of HDP outcomes between the two groups.

Our study has some limitations. First, while our study is retrospective and non-randomized in nature, it is important to recognize that randomized controlled trials (RCT) in this context are neither feasible nor ethical. Consequently, we were unable to eliminate potential confounding effects of "indications themselves" on the risk of through RCT design, which may affect the causal inference between donor sperm use and increased HDP incidence. Furthermore, information bias inherent in retrospective data collection cannot be fully avoided. Pre-pregnancy lifestyle factors (e.g., dietary patterns, physical activity intensity), psychological stress levels (related to infertility treatment or gamete donation), and detailed family medical history (e.g., grade of parental hypertension, onset age) were not fully captured in medical records, which may have introduced residual confounding. Secondly, donor-specific confounding factors cannot be completely ruled out. Potential biological differences between donor sperm and partner sperm (e.g., genetic polymorphisms related to placental development, epigenetic modifications) may contribute to HDP risk, but these factors were not analyzed in this study due to limited data availability. Thirdly, the study population was recruited from a single reproductive medical center, and the results may not be generalizable to broader populations. The racial/ethnic composition, regional healthcare practices, and donor sperm screening criteria in this study may differ from those in other countries or regions, further limiting the external validity of the findings. Finally, we did not analyze subtypes of HDP (e.g., gestational hypertension, preeclampsia, eclampsia) or long-term maternal and offspring outcomes (e.g., postpartum hypertension, cardiovascular disease risk in offspring), which limits the comprehensiveness of the findings.

Despite these limitations, this study has several strengths. Firstly, to our knowledge, it is one of the few studies focusing on the association between DS-IVF and HDP incidence, providing valuable real-world evidence for clinical practice. Secondly, we selected patients undergoing PS-IVF due to tubal factor infertility as the control group, which minimized the heterogeneity between DS-IVF and PS-IVF cycles as much as possible. Furthermore, the substantial sample size of included cycles ensured greater statistical power and credibility of the results.

Consequently, conducting animal studies is imperative and will help elucidate the pathophysiological mechanisms underlying the increased risk of HDP conceived with donor sperm and identify which specific component is responsible for immune tolerance, sperm or semen? Additionally, exploring pathophysiological studies of placenta from both donor and partner sperm IVF pregnancies is of critical importance. These researches might provide promising approaches for reducing, preventing, and treating hypertensive disorders associated with DS-IVF.

In conclusion, the use of donor sperm is an independent risk factor for new-onset HDP in IVF-conceived pregnancies. Among pregnancies conceived using donor sperm and partner sperm independently, higher BMI and twin pregnancy were independent risk factors for new-onset HDP.

## Data Availability

All data were available upon request.
